# Evaluation of the mandibular condyles trabecular structure in patients with severe class III pattern: a computed tomography (CT) fractal analysis study

**DOI:** 10.1038/s41598-023-45407-6

**Published:** 2023-10-24

**Authors:** Saeed Afzoon, Farhad Ghorbani, Mahvash Hasani

**Affiliations:** 1 Student Research Committee, Shiraz University of Medical Sciences, Shiraz, Iran; 2 Department of Oral and Maxillofacial Surgery, School of Dentistry, Shiraz University of Medical Sciences, Shiraz, Iran; 3 Department of Oral and Maxillofacial Radiology, School of Dentistry, Shiraz University of Medical Sciences, Shiraz, Iran

**Keywords:** Anatomy, Health care, Risk factors, Mathematics and computing

## Abstract

Class III malocclusion is a combination of dental and skeletal disorders that causes discrepancies in occlusion. Malocclusion can affect the structure of the Temporomandibular joint (TMJ) resulting in many problems, one of which is affecting the internal structure of the bone. This study aimed to examine the morphological features of class III patients’ trabecular structure of the mandibular condyle in comparison with normal class I individuals using fractal analysis (FA). To study the internal structure of the mandibular condyle bone, Computed Tomography (CT) scans of 45 severe class III patients (age: 16–46) who were the candidates for orthognathic surgery were selected and matched by age and sex with 45 normal class I individuals. The trabecular bone structure of the left and right mandibular condyles in three CT planes of the study group and control group were evaluated employing the FA. The result of the present study indicated that the fractal dimensions (FD) values of class III patients were lower than those of the normal class I individuals in axial (class I: 1.31 ± 0.02, class III: 1.28 ± 0.02), sagittal (class I: 1.25 ± 0.03, class III: 1.19 ± 0.08), and coronal (class I: 1.5 ± 0.06, class III: 1.45 ± 0.07) planes (P < 0.001). There were no significant differences between the FD values of the males and females. The intra-group evaluation revealed that there was no correlation between age and FD values. No evidence of laterality was found regarding the FD values of the right and left condyles. Given the noticeable differences between the FD values, it can be implied that severe class III malocclusion may affect the trabecular pattern of the cancellous bone of the mandibular condyle compared to class I individuals. Therefore, due to the altered trabecular structure, clinicians should be cautious when planning treatments for these patients.

## Introduction

The temporomandibular joint (TMJ) is a unique and complex structure that allows the mandible to move in different directions^1,2^.The TMJ is a synovial joint composed of a biconcave articulating disk, the articulating surface of the temporal bone, and the condyle of the mandible, enclosed in a fibrous capsule^3^. These parts allow the mandible to move in different directions and the TMJ to operate correctly^3,4^. Maintaining the health of the TMJ and its components is vital for having a well-functioning masticatory system^5^.

Class III malocclusion is a dental and skeletal condition that results in a concave profile due to mandibular prognathism, maxillary retrognathism, or a combination of the two^6–9^. The severity of this condition ranges from mild to severe, influencing the treatment planning with severe cases potentially requiring surgical intervention^7,10,11^. ClassIII malocclusion is a multifactoral disorder believed be caused as a result of interaction between genes and environment^7^. Treatment of malocclusions establishes a dynamic balance between different components of the masticatory system, enhances self-esteem, and takes the stress away from the craniofacial complex^12–14^.

Severe malocclusions can affect the internal and external structure of the bone^15^. Malocclusions can change the characteristics of the mandibular condyle, such as its hardness, the configuration of the trabecular meshwork, and the outer surface structure^15^.

Computed Tomography (CT) imaging techniques help us evaluate the bone structure effectively^16^. The CT scans provide precious information regarding the cortical borders and internal structure of the bone^17^. CT images aid us in studying the bone changes in three separate planes^18^.

Fractal analysis (FA) is a mathematical tool used to analyze the complexity of trabecular patterns^19–21^. It is used in many scientific disciplines to investigate complex and self-similar patterns^20^. Higher fractal dimensions (FD) demonstrate higher quality and complexity of bone internal structure^22^. The FD can be calculated on tomographic slices segmented at various threshold levels using the box-counting method^23^.

Some studies apply FA to measure the effect of transverse mandibular deficiency malocclusion, temporomandibular disorder, and rheumatoid arthritis on the mandibular condyle trabecular pattern^15,19,24^. Another study compares the FD values of the mandibular condyle trabecular pattern in class III, class II, and class I groups using two-dimensional (2D) radiographs^25^. To the best of our knowledge, the impact of the severe class III malocclusion on the microstructure of the mandibular condyle, in comparison to the typical class I subjects, using FA on CT images, has not been investigated to date. Therefore, the aim of the present study was to analyze the FD values of the mandibular condyles trabecular pattern in Class III patients and compare it to the matched control group (class I individuals) by investigating FD values on CT scans.

## Method and material

In this retrospective cross-sectional study, all methods were performed in compliance with the Helsinki Declaration. This study received approval from the Shiraz University of Medical Science Ethics Committee with the reference number IR.SUMS.DENTAL.REC.1401.069. Based on the procedures and protocols of Shaid Rajaee Hospital, informed consent had been obtained from all participants or their legal guardians before taking radiographic images.

To investigate the FD values of the trabecular pattern of the mandibular condyles, we extracted the CT scans from the Shahid Rajee Hospital archive. Initially, to form the study group, we selected the CT scans of 72 severe class III patients who required orthognathic surgery between 2018 and 2022. Subsequently, we included 45 subjects (22 female, 23 male) aged from 16 to 46 years in the study group. We formed the control group using the CT scans of the individuals who were referred to Shahid Rajaee Hospital for rhinoplasty or other CT scanning procedures. We ensured that individuals in the control group matched the age and sex of the study group. We carefully selected 45 class I subjects based on these requirements. Subjects in both groups needed to successfully pass the cephalometric analysis step, and meet the predefined inclusion criteria. Individuals with a history of diseases that affect bone metabolism, consuming drugs that affect bone turnover, trauma, any cyst or tumor, tooth loss except for third molar, or congenital abnormalities (e.g., cleft lip and palate) were excluded from the study. A maxillofacial radiologist with ten years of experience supervised the data extraction.

**Figure 1:**
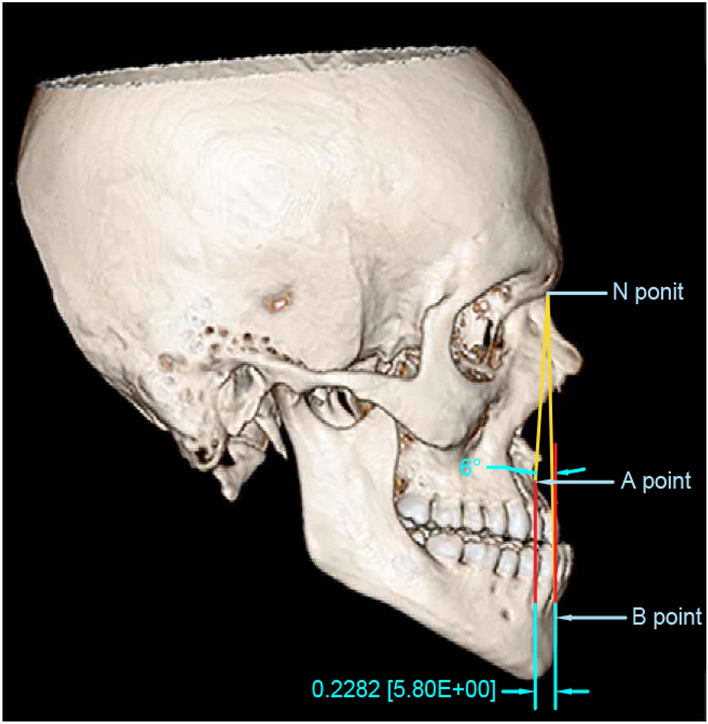
Cephalometric analysis on the 3D model using AutoCAD software.

Three-dimensional (3D) CT models were used for cephalometric analysis and they provided results similar to the two-dimensional (2D) cephalogram^26^. In order to satisfy the cephalometric criteria for both the study and control groups, A point-Nasion-B point (ANB) angle, Wits appraisal, and overjet were measured on the 3D CT models using AutoCAD software version 23 (Fig. 1). Patients with ANB angle < 0, Wits appraisals for females ≤ 0, for males ≤ − 1, and reverse overjet ≥ 3 were selected for the study group^27,28^. Individuals with ANB angle = 2° ± 2, Witts = − 1 mm for men and 0 mm for women, and overjet between 1.5 and 2.5 mm were classified as typical class I^27,29,30^.

**Figure 2:**
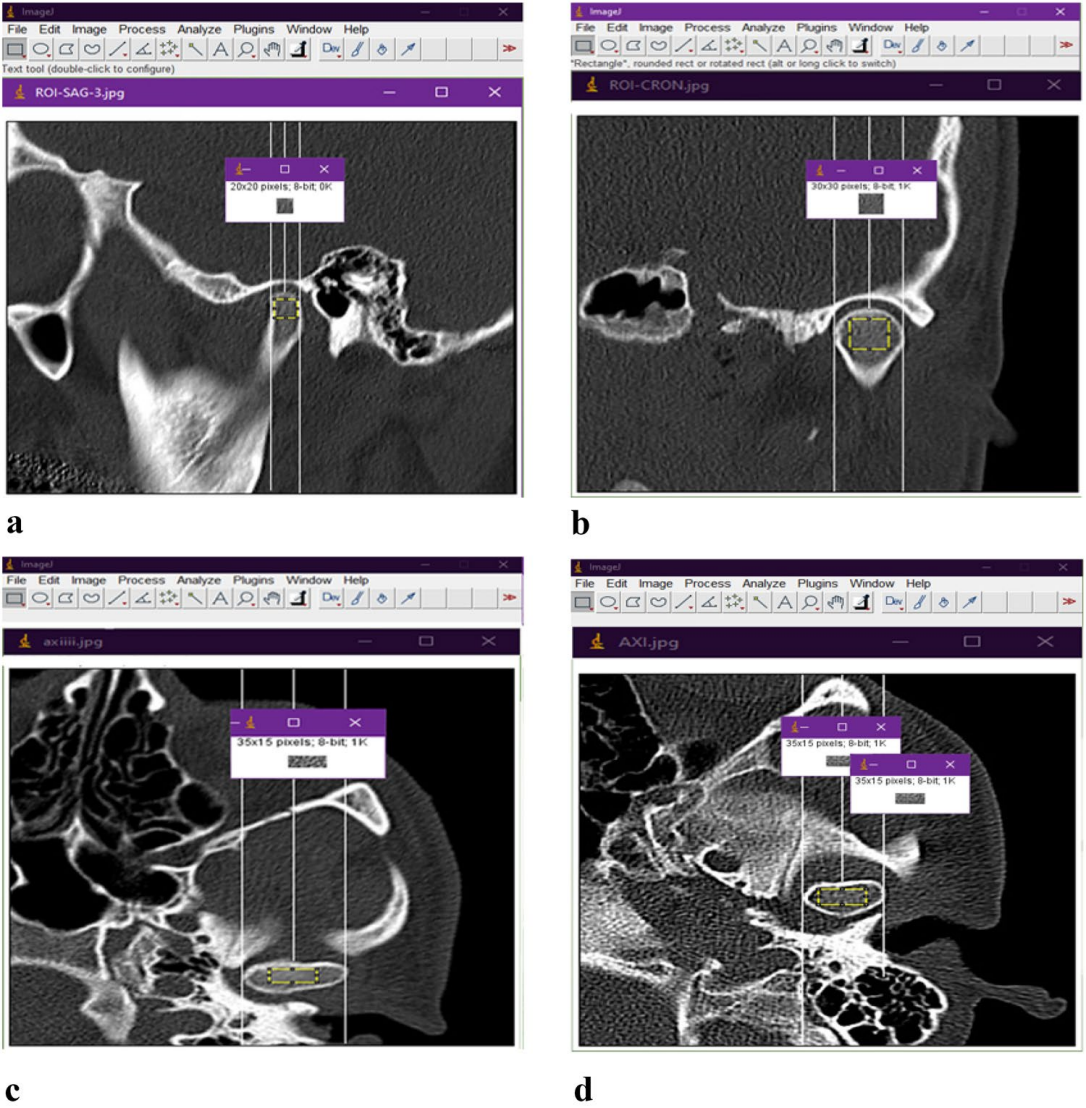
(**a**) Selection of the ROI in the sagittal plane, white perpendicular lines help us select the ROI from the middle and superior part of the condyles. (**b**) Selection of the ROI in the coronal plane, white perpendicular lines help us to select the ROI from the middle and superior part of the condyles. (**c**) Selection of the ROI in the axial plane, white perpendicular lines help us to select the ROI from the middle part of the condyles. (**d**) The ROI were selected and duplicated.

All CT scans were taken using a Siemens Emotion 16 (2010) CT scan device. The tube voltage (kVp) was set to 80 kVp, and the tube current–time product (milliampere-second or mAs) was set at 60 mAs. The voxel size, which determines the resolution of the CT images, was adjusted to 0.4824 × 0.4824 × 3.1748 mm^3^ and the slice thickness was set at 3.1748 mm. To perform the FA, initially, high-quality CT images were extracted in JPEG format. The analysis of the image was conducted on the ASUS laptop model X543MA equipped with Intel® Celeron® N4000 Processor 1.1 GHz (4M Cache, up to 2.6 GHz, 2 cores), graphic Intel UHD Graphics 600, RAM 4 GB and 500 GB Samsung SSD hard storage while operating on windows 10 pro 64 bit. The ImageJ software version 1.53 K (NIH, MD, USA) was downloaded from https://imagej.nih.gov/ij/download/ for image processing and measuring the FD values. Image processing was performed using the method designed by White and Rudolph^31^. The region of interest (ROI) was selected in three different views: In the axial view, a rectangular ROI (35 × 15 pixels), in the sagittal view a square ROI (20 × 20 pixels) in the coronal view a square ROI (30 × 30 pixels). The resolution of all ROIs corresponded to 2.2857 pixels per mm. We tried to select the ROIs from the middle and most superior part of the condyle in the sagittal (Fig. 2a) and coronal view (Fig. 2b) and from the middle of the condyle in the axial view (Fig. 2c). To select the ROIs, we first draw lines that pass through the lateral and medial borders of the condyles in the axial and the coronal view and through the anterior and posterior borders in the sagittal view. Then, in order to determine the center of the condyles more accurately, we draw a line in the middle of these lines.

**Figure 3:**
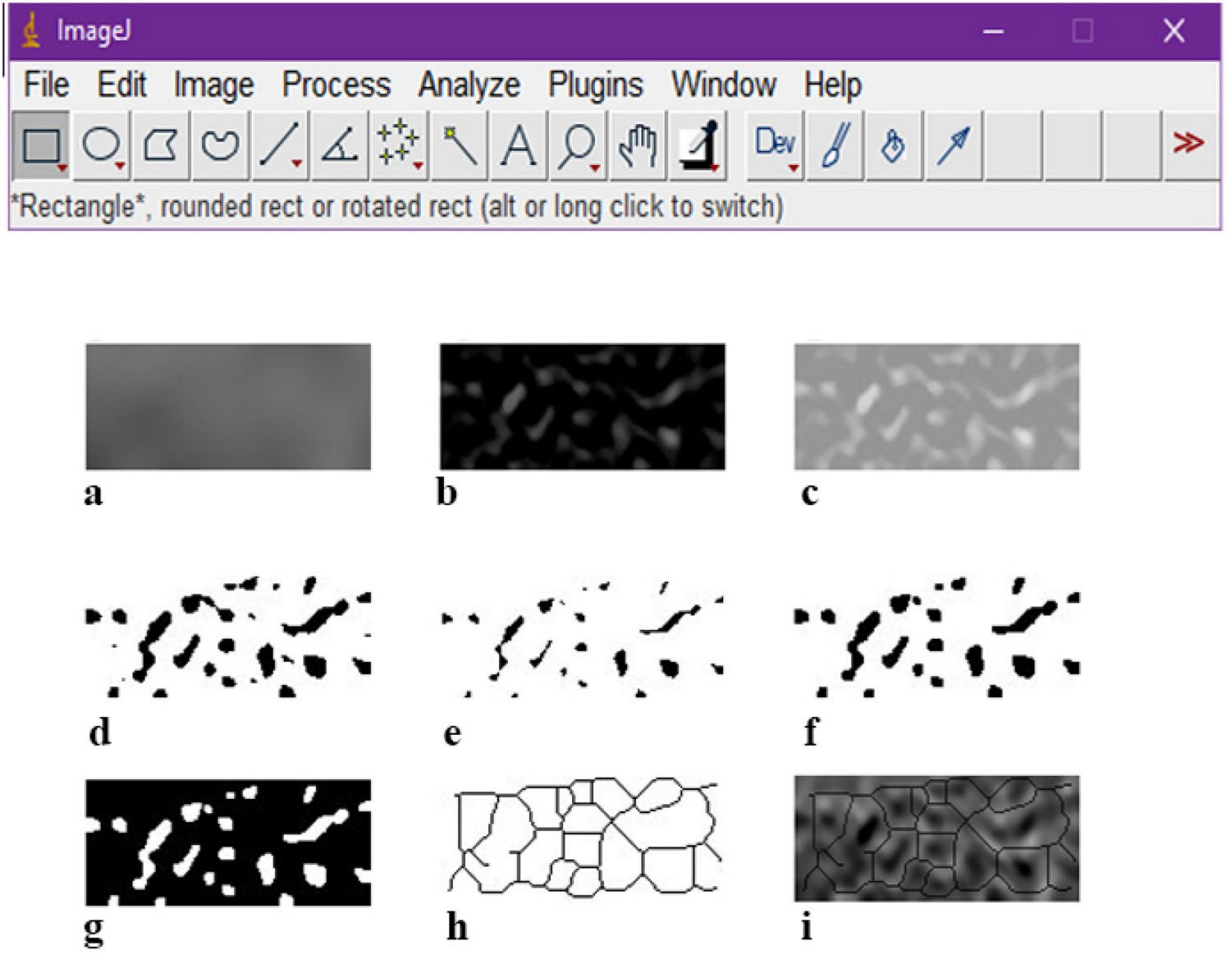
(**a**) Blurred, (**b**) Subtracted from the original, (**c**)128 value added, (**d**) Binarization, (**e**) Erosion, (**f**) Dilation, (**g**) Inversion, (**h**) Skeletonization, (**i**) Superimposition of the skeletonized image on the original image to show that it corresponds the trabecular structure.

The ROIs were selected, cropped, and duplicated (Fig. 2d). The duplicated images were blurred by applying the Gaussian blur filter to retain large variations in density (Fig. 3a). The blurred images were subtracted from the original image, to sharpen the image and make the edges prominent (Fig. 3b). 128 value was added to every pixel to shift the brightness level upward (Fig. 3c). The image then was binarized, eroded, dilated, inverted, and skeletonized in sequence (Fig. 3d–h). Erosion and Dilation tools were applied consecutively to reduce the noise of the image. In the skeletonization step, the trabeculae were transformed into a line and became ready for FA. To confirm that the skeletonized image represents the structure of the trabeculae, we superimposed the skeletonized image on the first image to show that the trabecular pattern of the skeletonized image corresponded to the original image (Fig. 3i).

**Figure 4:**
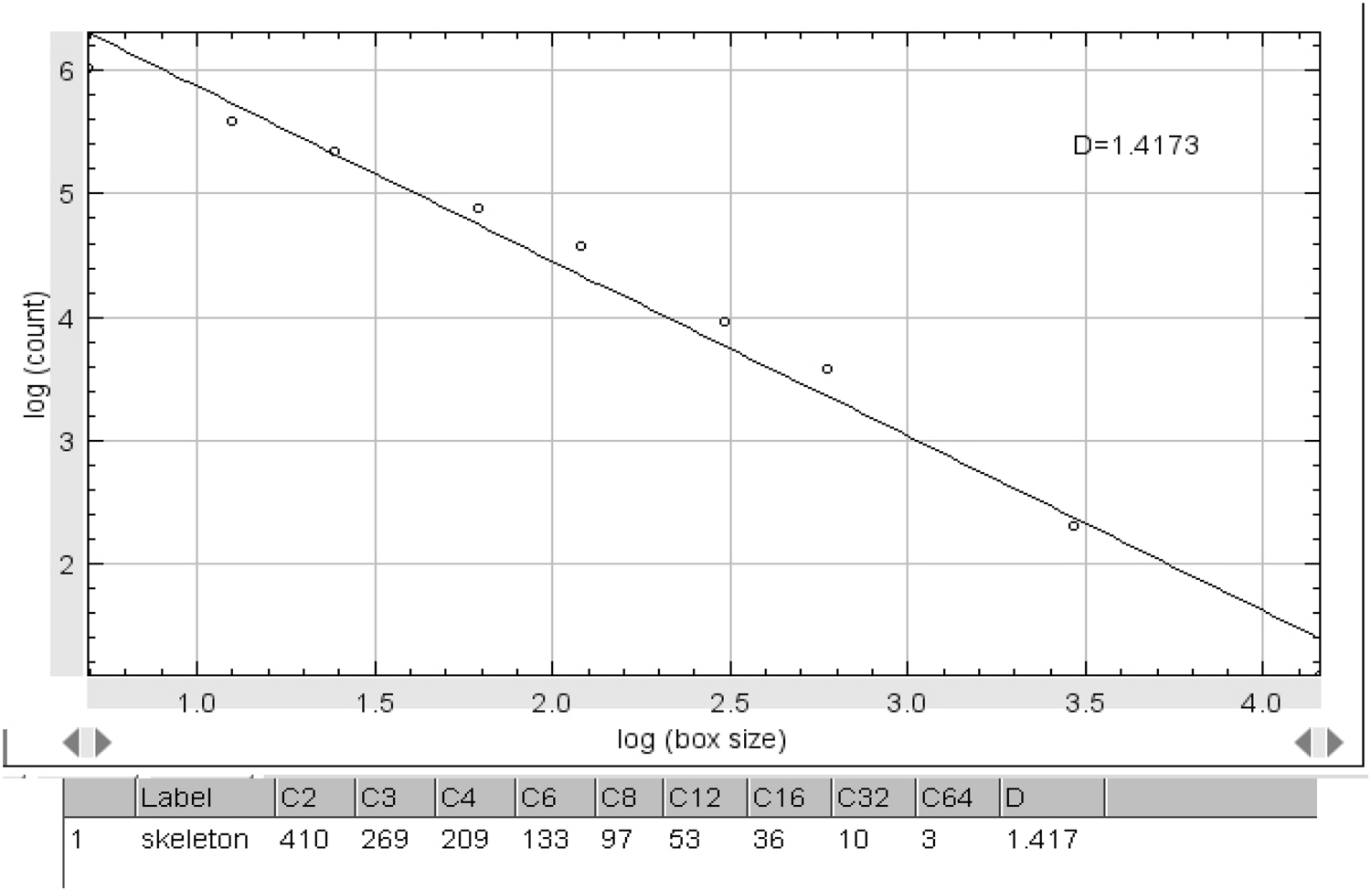
The slop of the line indicates the FD value.

The FD values of the skeletonized images were calculated using the ImageJ box-counting algorithm from the “Analyze” menu. In the box-counting method, the images are divided into the squares of 2, 3, 4, 6, 8, 12, 16, 32, and 64 pixels and for each pixel size, the number of boxes including trabeculae was calculated, and plotted against the box sizes on a logarithmic scale graph. The slope of the line on the graph represented the FD which indicated the degree of complexity of the trabeculae architecture (Fig. 4).

FA was performed and supervised by a skilled oral and maxillofacial radiologist. The same radiologist reexamined 80 pictures of 20 randomly chosen patients in coronal, axial, and sagittal planes to evaluate the validity of ROI selection. One month following the original assessment, the CT scans were examined to assess the intra-observer reliability.

### Statistical analysis

All statistical analyses were performed using SPSS software version 25 (IBM Corp, Armonk, NY, USA). Each variable was summarized using frequency and percentage or median (IQR) and mean ± SD. FD in the form of a continuous variable was the outcome variable of interest. The predicator variables of interest included age, gender, type of occlusion, and malocclusion. Age and fractal dimension were introduced as continuous variables, while other predictor variables were reported as categorical variables. The normality of the distribution of the data was tested by a one-sample Kolmogorov–Smirnov test. Since the measurements were not normally distributed, the FD values of three different planes (axial, sagittal, and coronal) were compared between the study and control groups using the Mann–Whitney *U* test. The comparison of the FD values of the left and right condyle within and between the study and control groups was also carried out using the Wilcoxon rank sum test. The correlation of the FD values and age in each group in three different CT scan planes was evaluated using Spearman's rank correlation. The intraclass correlation coefficient (ICC) was calculated to evaluate the intra-observer reliability of the FD values measurement twice in 1-month intervals. To test statistical power, we have run post hoc power analysis utilizing G*Power 3.0.10.

## Results

As we used perpendicular lines guiding us to select the ROI, we observed an excellent correlation between the repeated fractal dimension measurements with an average of 0.982 (95% CL 0.972–0.988) affirming reliable intra-rater reliability. Data for a total of 45 (23 males, 22 females) class III patients were collected. To eliminate the effect of age and gender, the control group was formed consisting of 45 people based on the gender, age, and frequency of the study group. The average age in both groups was 23.44 years old (51.1% male and 48.9% female).

The comparison of the FD values between the case (class III patients) group and control (class I individuals) group in three different planes (sagittal, axial, and coronal) was presented in Table 1. The results showed that in all three planes, the FD values of the control group were significantly higher than those of the study group (P < 0.001).

**Table 1:** Comparison of the FD values between case and control groups in three plans of CT scans.

Planes	Group		P-value^a^
	Case (n = 45)	Control (n = 45)	
Saggital	1.19 ± 0.07	1.25 ± 0.03	< 0.001
	1.2 (1.14–1.24)	1.25 (1.22–1.27)	
Axial	1.28 ± 0.02	1.31 ± 0.02	< 0.001
	1.28 (1.26–1.30)	1.31 (1.30–1.33)	
Coronal	1.45 ± 0.07	1.5 ± 0.06	< 0.001
	1.47 (1.39–1.51)	1.52 (1.48–1.54)	

Values reported by Mean ± SD and Median (IQR).

^a^Mann–Whitney *U* test.

**Figure 5:**
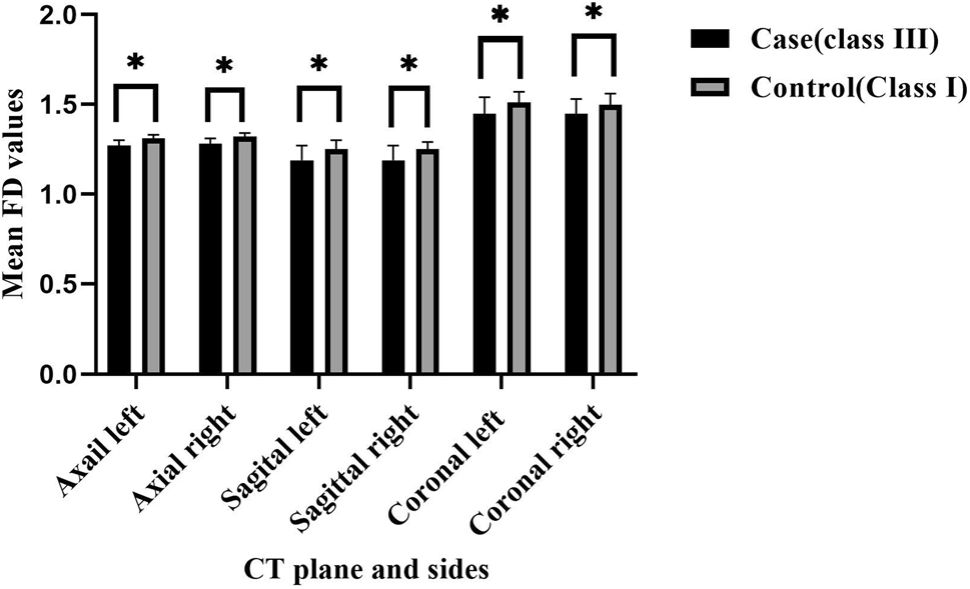
Comparison of FD values of the left and right condyle within and between the case and control groups in three plans of CT scans.

The intergroup comparison of the left and right condyles of the study group with their matched condyles in the control group revealed that the FD values of the study group were significantly less than those of the control group in all three planes (P ≤ 0.001). In addition, within each of the study and control groups, no statistically significant differences between the left and right condyles were found (p > 0.05). The results of the comparison of the FD values of the left and right condyle, both within and between the case and control groups, in three planes of CT scans were outlined in Table 2 and visually represented in Fig. 5.

**Table 2:** Comparison of FD values of the left and right condyles within and between the case and control groups in three plans of CT scans.

Planes	Side	Group		P-value^a^
		Case (n = 45)	Control (n = 45)	
Sagittal	Left	1.19 ± 0.08	1.25 ± 0.05	P < 0.001
		1.21 (1.14–1.25)	1.26 (1.22–1.29)	
	Right	1.19 ± 0.08	1.25 ± 0.04	P < 0.001
		1.19 (1.11–1.25)	1.25 (1.21–1.27)	
	P-value^b^	0.88	0.71	
Axial	Left	1.27 ± 0.03	1.31 ± 0.02	P < 0.001
		1.28 (1.26–1.3)	1.31 (1.29–1.33)	
	Right	1.28 ± 0.03	1.32 ± 0.02	P < 0.001
		1.29 (1.27–1.3)	1.31 (1.3–1.33)	
	P-value^b^	0.19	0.17	
Coronal	Left	1.45 ± 0.09	1.51 ± 0.06	0.001
		1.48 (1.39–1.52)	1.53 (1.48–1.54)	
	Right	1.45 ± 0.08	1.5 ± 0.06	P < 0.001
		1.48 (1.4–1.51)	1.52 (1.5–1.54)	
	P-value^b^	0.95	0.72	

Values reported by Mean ± SD and Median (IQR).

^a^Mann–Whitney *U* test.

^b^Wilcoxon rank sum test.

The comparison between the FD values of the male and female individuals was conducted within the case and the control groups for all three planes of the CT scans and presented in the Supplementary Table S1 online. The results indicated no statistically significant difference between the two gender groups (P > 0.05). Additionally, the correlation of the age with the FD values in each group was also investigated and presented in the Supplementary Table S2 online. The results indicated a lack of correlation between the two variables (P > 0.05).

The effect sizes were calculated using the mean and standard deviations of the FD values in three CT scan planes to test the statistical power. The observed power of the two-tailed tests was estimated to be very good for all three planes (sagittal = 0.99, axial = 1, and coronal = 0.94) with a 5% alpha significant.

## Discussion

FA is a widely used method to evaluate the complex geometric internal microstructures of the bone, providing useful information about its density and strength^32^. In dentistry, it has been employed to study the systemic disease affecting the TMJ, bone healing around implants, and the effect of periodontal disease on the bone microstructure^15,19,33–35^. In orthodontics, it is used to investigate the midpalate suture activity, to evaluate the effect of orthodontic treatment duration on the mandibular bone, to assess the bone microstructure, and to examine the bone around the impacted maxillary canines^36–39^. Moreover, FA has been used to evaluate the effect of malocclusion on the mandibular condyles using 2D images and it has been employed to examine the impact of the mandibular transverse deficiency on the internal structure of the condyles using cone-beam computed tomography (CBCT) images^15,25^. The FA technique also helps us distinguish osteoporotic patients from healthy individuals^40^. Similarly, texture analysis is a method which is used to evaluate the intensity, color, and brightness of the images and provides quantitative data to investigate the features of images^41^. The result of the texture analysis similar to the FA can be incorporated into the clinical practice^42^. However, no study has evaluated the effect of the class III malocclusion on the mandibular condyle microstructure using CT images employing FA.

CT images offer higher resolution and greater detail of the trabecular pattern of the bones, allowing for a more precise evaluation of the structure and morphology of the mandibular condyle trabeculae^43^. Radiographic images help us quantify the microarchitecture of trabeculae and measure the FD values using various methods^32^. The present study evaluated the FD values of the mandibular condyles using the box-counting method^44^. This technique counts the number of boxes that include some trabeculae, calculates the size of the boxes, and then plots the data on a logarithmic scale graph to determine the FDs^44^.

In a study on three different dentofacial skeletal patterns, Korkmaz and Arslan discovered no significant differences between the right and left condyles FD values^25^. They noticed that the FD values of the right condyle were higher than those of the left condyle, although this difference was not statistically significant^25^. They concluded that it might be due to the individuals' tendency for right-side dominance during oral activities^25^. In contrast to their result, our results did not support the laterality.

Several studies have examined the impact of the age on the FD values, as evidenced by the literature^15,19,25,41^. In this study, we did not find any correlation between the age and the FD values of the mandibular condyle. In this regard, Korkmaz and Arslan have proposed that the FD value gradually declines in Class III patients as the condyles grow continuously^25^. Furthermore, they have argued that longitudinal evaluation of the FD values of the condyles in class III patients is necessary to monitor any potential changes that may occur in the internal trabecular structure of the condyles over time^25^.

Regarding the correlation of the sex with the FD values, Korkmaz and Arslan reported that class II division I female subjects had lower FD values than their male counterparts^25^. However, they did not discover any statistically significant differences between the FD values of males and females in class I, class II division II, and class III groups^25^. In this study, we did not find significant differences between the FD values of the male and female subjects in the study and control groups.

According to our findings, the FD values of the severe Class III patients were less than Class I individuals. Korkmaz and Arslan evaluated the FD values of Class III, Class II, and Class I malocclusions on panoramic images^25^. According to their research, Class III patients had the lowest FD values, whereas Class I participants had the highest^25^. Similarly, our results suggested that class III patients have lower FD values than class I participants. In this regard, Kocak and Bulut used FA on the CBCT images to assess the effects of the maxillary transverse deficiency, which results in malocclusion, and they discovered that the FD values of the study group right condyles were more than those of the control group^15^. Their finding, the same as our finding, suggests that malocclusion can affect the FD values of the mandibular condyle.

FA may be employed to evaluate the density and strength of the bone^45^. According to Lin et al.'s research, BMD and FD values both serve as indicators of bone strength; however, FA can study the biomechanical characteristics of the bone in more detail^46^. Examining the complexity of trabecular structure through FA is a quantitative method to evaluate the bone strength^32,47^. Lower FD value is associated with weaker bone, which may be the result of the changes in the internal bone structure and trabecular configuration^32,48,49^. Collaborating our results with the mentioned studies results, we can argue that class III patients may have weaker mandibular condyles than Class I subjects as they have lower FD values.

The direction of stress and efficiency of forces in the TMJ region is not the same in individuals with different skeletal patterns^50,51^. Malocclusion can alter the structure of the TMJ and it can result in excessive strain on its components^52,53^. Bae et al. reported that class III subjects have lower masticatory force efficiency compared to class I and class II individuals^51^. On the other hand studies have suggested that different force patterns delivered to the bone could alter the structure of the trabeculae^48,54^. Our findings reveal that the trabecular patterns of mandibular condyle in class III patients are less complex than class I individuals with normal forces and strain on the TMJ.

Forces and loads generated by orthodontic appliances can alter the trabecular structure^52,55^. According to Köse et al., the length of the orthodontic therapy might alter the FD values, and studies have demonstrated that class II patients' FD values changed after receiving treatment with functional appliances^52,55^. Forces on the bone can alter the trabecular pattern of the bone^54^. Additionally, based on our results, class III patients’ FD values were lower than those of typical class I subjects. These findings imply that clinicians should consider the likelihood that loads and strains from orthodontic equipment placed on the TMJ may change the trabecular architecture of the bone.

Orthognathic surgery can alter the structure of the trabeculae, in this regard, Kang et al. reported that the FD values decrease 1-month post-surgery^56^. Our study examined the CT images before the surgery and found that the trabecular structure of the Class III patients’ mandibular condyle is less complex than typical Class I patients. Additionally, it has been discussed before that lower FD values are related to lower bone strength^32,48,49^. This information underscores the importance of being cautious in applying loads on the TMJ region post-orthognathic surgery, given the potential changes in trabecular structure and implications for bone strength.

## Limitations

Only one image from each plane—sagittal, axial, and coronal—was used in this work to examine the mandibular condyle microstructure. Even though this method made it possible to evaluate the trabecular structure in various planes, future research should also consider the evaluation of the structure of trabeculae in 3D blocks of CT images rather than just using one image from each plane. This may help to better understand the impact of severe class III malocclusion on the bone quality and provide a more detailed portrayal of the internal microstructure of the mandibular condyle. Finally, this study examined the internal structure of the bone at a single point in time; however, longitudinal studies following the same patients over time help us better evaluate the internal trabecular pattern and its structural change.

## Conclusion

FA proves to be a valuable tool for assessing the trabecular pattern changes in severe Class III patients. The findings demonstrated that individuals with severe Class III malocclusion exhibited less complex trabecular patterns when compared to the typical Class I individuals, as revealed through FA on CT images.

### Supplementary Information

Supplementary Tables.

## Data Availability

Due to the regulations imposed by Shahid Rajaee Hospital, the data supporting the finding of this study is not publicly available. However, we would like to emphasize that the datasets used and analyzed during the current study are available from the corresponding author upon reasonable request.
