# Supplementary material for: Evaluation of the mandibular condyles trabecular structure in patients with severe class III pattern: a computed tomography (CT) fractal analysis study
Source: Sci Rep. 2023 Oct 24;13:18183. doi: 10.1038/s41598-023-45407-6 (PMC10598208; doi:10.1038/s41598-023-45407-6)
Supplement: Supplementary file 1 — Supplementary Tables. [file 41598_2023_45407_MOESM1_ESM.pdf]

Evaluation of the Mandibular Condyles Trabecular Structure in Patients with Severe Class III Pattern: A Computed Tomography (CT) Fractal Analysis Study

Saeed Afzoon, Farhad Ghorbani, Mahvash Hasani

Supplementary Table S1: comparison of FD values between male and female individuals in three different plans of CT scans within case and control groups

Planes	group					
	case			control		
	Male	Female	p-value[a]	Male	Female	p-value[a]
sagittal	1.17 ± 0.07<br>1.18 (1.12-1.23)	1.2 ± 0.05<br>1.21 (1.15-1.25)	0.2	1.24 ± 0.03<br>1.25 (1.22-1.26)	1.25 ± 0.03<br>1.25 (1.23-1.28)	0.63
axial	1.28 ± 0.02<br>1.29 (1.26-1.3)	1.27 ± 0.02<br>1.27 (1.26-1.29)	0.29	1.32 ± 0.02<br>1.32 (1.31-1.33)	1.31 ± 0.02<br>1.31 (1.3-1.32)	0.75
Coronal	1.44 ± 0.07<br>1.45 (1.36-1.50)	1.46 ± 0.07<br>1.48 (1.42-1.52)	0.22	1.5 ± 0.07<br>1.52 (1.5-1.54)	1.51 ± 0.04<br>1.52 (1.48-1.54)	0.1

Values reported by Mean±SD and Median(IQR)
a: Mann-Whitney U test

Supplementary Table S2: correlation between FD values and age in three different plans of CT scans within case and control groups

Planes	group			
	case		control	
	r	p-value	r	p-value
sagittal	0.09	0.54	0.19	0.18
axial	0.24	0.1	-0.07	0.64
Coronal	0.25	0.09	0.12	0.4

r: Spearman correlation coefficient
